# Circulating total and H-specific GDF15 levels are elevated in subjects with MASLD but not in hyperlipidemic but otherwise metabolically healthy subjects with obesity

**DOI:** 10.1186/s12933-024-02264-5

**Published:** 2024-05-18

**Authors:** Chrysoula Boutari, Konstantinos Stefanakis, Stamatia Simati, Valentina Guatibonza-García, Laura Valenzuela-Vallejo, Ioanna A. Anastasiou, Margery A. Connelly, Alexander Kokkinos, Christos S. Mantzoros

**Affiliations:** 1 Department of Medicine, Beth-Israel Deaconess Medical Center, Harvard Medical School, 330 Brookline Ave, SL418, Boston, MA 02215 USA; 2 First Department of Propaedeutic Internal Medicine, Medical School, National and Kapodistrian University of Athens, Laiko General Hospital, Athens, Greece; 3 Labcorp, Morrisville, NC 27560 USA; 4 Department of Medicine, Boston Medical Center, Boston University School of Medicine, Boston, MA 02218 USA; 5 Department of Medicine, Boston VA Healthcare System, Boston, MA 02130 USA

**Keywords:** Growth differentiation factor 15, GIP, C-peptide, Non-alcoholic fatty liver disease, Obesity, Mixed meal test, Oral glucose tolerance test

## Abstract

**Background:**

Growth differentiation factor 15 (GDF15) is a mitokine, the role of which, total or H-specific, in modulating energy metabolism and homeostasis in obesity-related diseases, such as metabolic dysfunction associated steatotic liver disease (MASLD), has not been fully elucidated in adult humans. We aimed to investigate the fasting and stimulated levels of GDF15, total and H-specific, glucose-dependent insulinotropic polypeptide (GIP) and C-peptide, in two physiology interventional studies: one focusing on obesity, and the other on MASLD.

**Methods:**

Study 1 investigated individuals with normal weight or with obesity, undergoing a 3-h mixed meal test (MMT); and study 2, examined adults with MASLD and controls undergoing a 120-min oral glucose tolerance test (OGTT). Exploratory correlations of total and H-specific GDF15 with clinical, hormonal and metabolomic/lipidomic parameters were also performed.

**Results:**

In study 1, 15 individuals were included per weight group. Fasting and postprandial total and H-specific GDF15 were similar between groups, whereas GIP was markedly higher in leaner individuals and was upregulated following a MMT. Baseline and postprandial C-peptide were markedly elevated in people with obesity compared with lean subjects. GIP was higher in leaner individuals and was upregulated after a MMT, while C-peptide and its overall AUC after a MMT was markedly elevated in people with obesity compared with lean subjects. In study 2, 27 individuals were evaluated. Fasting total GDF15 was similar, but postprandial total GDF15 levels were significantly higher in MASLD patients compared to controls. GIP and C-peptide remained unaffected. The postprandial course of GDF15 was clustered among those of triglycerides and molecules of the alanine cycle, was robustly elevated under MASLD, and constituted the most notable differentiating molecule between healthy and MASLD status. We also present robust positive correlations of the incremental area under the curve of total and H-specific GDF15 with a plethora of lipid subspecies, which remained significant after adjusting for confounders.

**Conclusion:**

Serum GDF15 levels do not differ in relation to weight status in hyperlipidemic but otherwise metabolically healthy individuals. In contrast, GDF15 levels are significantly increased in MASLD patients at baseline and they remain significantly higher compared to healthy participants during OGTT, pointing to a role for GDF15 as a mitokine with important roles in the pathophysiology and possibly therapeutics of MASLD.

*Trial registration* ClinicalTrials.gov NCT03986684, NCT04430946.

**Supplementary Information:**

The online version contains supplementary material available at 10.1186/s12933-024-02264-5.

## Background

Metabolic dysfunction-associated steatotic liver disease (MASLD), also known as non-alcoholic fatty liver disease (1) is the most common liver disease, which is present in approximately a third of the global population, and it is strongly associated with insulin resistance and metabolic syndrome [1, 2]. MASLDis characterized by more than 5% liver fat accumulation, and it represents a spectrum of histological characteristics ranging from fat deposition in the hepatocyte to inflammation and hepatocyte damage, known as metabolic dysfunction-associated steatohepatitis (MASH) [3]. MASH could lead to complications including liver fibrosis/cirrhosis, hepatocellular carcinoma, and cardiovascular disease [2]. Although MASLD is an emerging public health concern [4–6], at this time, there is only one, very recently U.S. Food and Drug Administration (FDA) approved, pharmacological treatment, resmetirom, which is a thyroid hormone receptor-beta (THR-beta) agonist [7, 8]; thus, lifestyle modification with caloric-restrictive diets, exercise, weight loss, and the management of the components of metabolic syndrome are the mainstay of treatment for MASLD [9]. Additionally, the gold standard for the diagnosis of MASLD involves liver biopsy, an invasive, expensive, and risky procedure, and there is a lack of non-invasive, accurate, diagnostic tools [10, 11]. Thus, there is an unmet clinical need regarding understanding the underlying pathophysiology and developing novel therapeutic and diagnostic tools for MASLD. Of note, recently a modified Delphi process and several voting rounds in which academic professionals and experts from around the world participated in, were conducted by the American Association for Study of Liver Disease (AASLD), the European Association for Study of the Liver (EASL), and the Asociación Latinoamericana para el Estudio del Hígado (ALEH) and led to a change in the nomenclature and the diagnostic criteria for the disease. The Metabolic Dysfunction-Associated Steatotic Liver Disease (MASLD), replaced the old, nonspecific, and even stigmatizing due to the terms “alcohol” and “fat”, MASLD terminology [12, 13].

Growth differentiation factor 15 (GDF15) (previously known as macrophage inhibitor cytokine-1) is a secretory protein that belongs to the transforming growth factor beta (TGFβ) superfamily. It is expressed in the liver, skeletal muscle, adipose tissue, kidney, heart, placenta and in macrophages, among others [14]. GDF15 increases in response to various intra- [15, 16] and extra- [17, 18] cellular stress-related conditions, such as mitochondrial or endoplasmic reticulum (ER) defects, energy/overload states, or infections. In addition, its increase is associated with MASLD, heart failure [19], chronic kidney disease (CKD) [20], pulmonary fibrosis [21], sepsis [18], and cancer [22], as it has been demonstrated in either animal models or humans and may have therapeutic or biomarker potential that needs to be studied further [23]. However, the role of GDF15 in humans remains to be fully elucidated.

In animals, GDF15 knockout mice exposed to high-fat diet are more likely to become obese and to have impaired glucose tolerance and lower metabolic rate [24]. In contrast, other studies suggested that GDF15 administration to obese mice resulted in body weight reduction by decreasing food intake [25]. Of note, it has been recently suggested that GDF15 enhances body weight and adiposity reduction in obese mice by stimulating the leptin pathway [26]. Data in humans remain controversial. Although some studies have shown a relationship between GDF15 and obesity and thus proposed a role in modulating energy metabolism and homeostasis [27], others have casted doubts on these findings [28, 29]. In MASLD, GDF15 has been studied in animals and humans [30–34].

In addition, many other peptides have been linked with MASLD. In the past, we have shown, through a targeted hormonal investigation, the differential regulation of GLP-1, activins/follistatins, insulin, and insulin growth factors across a wide range of MASLD pathologies [35]. However, we had not assessed levels of glucose-dependent insulinotropic polypeptide (GIP) and C-peptide, whereas the role of GDF15 remains to be elucidated. GIP is an incretin hormone, released by enteroendocrine K-cells in response to glucose intake, and is an important mediator of the early insulin response [36]. C-peptide is one of the segments of proinsulin, representing endogenous insulin secretion by islet β cells and insulin resistance [37], and it has been shown to be significantly associated with MASLD [38].

To further understand the role of GDF15 in the pathophysiology of obesity-related human diseases and to clarify whether GDF15 levels are associated with MASLD, we investigated circulating total and H-specific GDF15 levels in humans with obesity or MASLD at baseline as well as their changes during a mixed-meal test (MMT) or an oral glucose tolerance test (OGTT), respectively. Additionally, we estimated the corresponding levels of GIP and C-peptide and their response after a meal or an oral glucose load. Furthermore, we utilized a multi-omics approach to explore the potential implications of GDF15 in altering metabolic pathways that could contribute to the pathogenesis of MASLD.

## Methods

### Study 1: physiology interventional study assessing the postprandial levels of GDF15, GIP, and C-peptide, in individuals with normal-weight versus with obesity following a 600-kcal mixed meal test

This study (Study 1) (NCT04430946) aimed to assess primarily the fasting and postprandial levels of GDF15, and secondarily C-peptide, and GIP in individuals with absence of metabolic disease other than obesity, compared with a control group of normal weight participants. A one-arm, interventional study was implemented in apparently healthy (free of type 2 diabetes and other obesity-related comorbidities and with minimal fluctuations in body weight [< 5 kg] in the 3 months prior to study initiation) individuals with normal weight or otherwise healthy individuals with obesity. Participants were voluntarily recruited from the outpatient clinics of the 1st Department of Propaedeutic Internal Medicine, Laiko General Hospital, University of Athens, Greece. In brief, fifteen individuals were recruited per weight group and included in the current analysis. The two groups were age- and sex- matched and were directly compared in terms of their response to the same intervention (see below). The intervention was a high-fat mixed meal containing 600 kcal (80% fat, 19% protein, 1% carbohydrates) following an overnight fast. Inclusion criteria were set as follows: age 18–65; body mass index (BMI) ≥ 30 kg/m^2^ (individuals with obesity); BMI ≤ 25 kg/m^2^ (normal-weight individuals); body weight fluctuation < 5 kg in the last 3 months. Exclusion criteria were set as follows: uncontrolled hyper- or hypothyroidism; gastrointestinal disorders leading to malabsorption.

### Study 2: pathophysiology interventional case–control study assessing the postprandial levels of GDF15, GIP, and C-peptide, in individuals with early stage MASLD and healthy controls undergoing a 75 g OGTT

This study (Study 2) (NCT03986684) aimed to investigate the fasting and postprandial levels of GDF15, C-peptide, and GIP in individuals with early stage MASLD compared with healthy participants. The design and methods have been previously described in detail [35]. Adult participants were voluntarily recruited from the outpatient clinics of the Second Propaedeutic Department of Internal Medicine, Ippokration Hospital, Aristotle University of Thessaloniki, Greece. Thirty-two individuals were enrolled, with 27 being included in the current analysis (5 participants excluded due to limited amount of serum) and were divided into healthy controls and MASLD patients on the basis of ultrasound and fatty liver index (FLI). FLI is a non-invasive clinical score indicating the likelihood of fatty liver disease [FLI = (e0.953  * loge (triglycerides) + 0.139 * BMI + 0.718 * loge (GGT) + 0.053 * waist circumference − 15.745)/(1+e0.953 * loge (triglycerides) + 0.139 * BMI + 0.718 * loge (GGT) + 0.053 * waist circumference − 15.745) × 100], < 30 or FLI < 60 and normal liver ultrasound imaging [39]. In the MASLD group, we included patients with an FLI ≥ 30 and ultrasound imaging indicating fatty liver, based on the following ultrasound parameters: parenchymal brightness, liver-to-kidney contrast, deep beam attenuation, bright vessel walls, and gallbladder wall definition. Qualitative grades were conveniently labeled mild, moderate, or severe or grade 0 to 3 (with 0 being normal) [40]. People with FLI ≥ 60 and fibrosis-4 score (FIB-4) > 3.25, any secondary cause of fatty liver, alcoholic or drug-induced hepatitis, and viral or autoimmune hepatitis were excluded. All participants underwent a 75-g OGTT following an overnight fast, wherein blood samples were collected immediately before glucose administration and every 30 min postprandially, for up to 120 min. In addition, vital signs (heart rate, blood pressure, temperature), and anthropometric measurements were conducted.

It is worth mentioning that both studies are interventional studies, but also have a cross-sectional component as well. Specifically, they utilized acute interventions (subjects in study 1 underwent MMT and participants in study 2 underwent a 75-g OGTT) rather than prolonged treatments.

### Clinical and biochemical measurements

Anthropometric data were measured in the fasting state, in the morning during all visits. Body weight was measured without shoes, and in light clothing, and patient BMI was calculated.

In both studies, after anthropometric assessment, an intravenous cannula was placed in the forearm of each participant before meal consumption and 20 ml of venous blood was drawn immediately before the meal and at 30-min intervals (for a total of 180 min in study 1; and 120 min in study 2). Four ml-EDTA tubes preprepared with the addition of Aprotinin 100 KIU/ml (Nordic Pharma Ltd, Reading, UK) were used for whole blood collection. Blood samples were centrifuged immediately at 4000 rpm at 4 °C in order for separated serum to be obtained. Following centrifuging, tubes were stored at − 80 °C.

Serum fasting glucose, insulin (Study 2 only), HbA1c, lipids, and liver enzymes, were measured using conventional hospital equipment. No patient had a known history of diabetes mellitus, whereas at baseline only 2 patients from Study 2 had levels of HbA1c > 6%

### Assays

Total GDF15, H-specific GDF15 (H2O2D variant non-detectable), and C-peptide were measured using two novel enzyme-linked immunosorbent assays (ELISA) (Ansh Labs LLC, Webster, TX, USA) as previously described [28]. GIP was measured by ELISA (Mercodia, Uppsala, Sweden).

### Omics measurements

For study 2, metabolomics and lipidomics analysis were measured with nuclear magnetic resonance spectroscopy (NMR) by Labcorp (Morrisville, USA). The complete list of the biomarkers measured and used for the -omics analysis can be found in Supplementary Table 1. NMR spectra were acquired on a Vantera® Clinical Analyzer, a 400 MHz NMR instrument, from fasting EDTA plasma samples as described for the NMR LipoProfile® test (Labcorp, Morrisville, NC) [41]. The LP4 deconvolution algorithm was used to report lipoprotein particle concentrations and sizes, as well as concentrations of metabolites such as total branched-chain amino acids, valine, leucine, and isoleucine, alanine, glucose, citrate, glycine, total ketone bodies, β-hydroxybutyrate, acetoacetate, acetone [42–47]. For more details on omics measurements see Supplementary Material.

### Statistical analysis

Statistical analysis was performed with SPSS Version 28.0 (IBM Corp., Armonk, NY), MetaboAnalystR (v. 6.0), Rstudio 2023.12.0 (Posit) using R v. 4.3.1 (The R Foundation), and Prism 9.3.1. (GraphPad Software Inc, La Jolla, CA). All variables were assessed for normality using the Shapiro–Wilk test; results are presented in tables as means ± SD in case of normally distributed variables, or interquartile ranges for non-normally distributed variables. Figures implement mean values ± standard errors of the mean to facilitate readability. An independent samples *t*-test/Mann Whitney *U* test depending on normality was performed for baseline characteristics comparisons, followed by ANCOVA to adjust for covariates. To analyze the changes in total and H-specific GDF15, GIP, and C-peptide levels in controls and MASLD during the OGTTs or MMTs a mixed-effects model was fitted, matching for subjects and assigning factors time (MMT or OGTT minutes) and group as repeated measures, with compound symmetry set as repeated covariance type. Time, group, and time * group were set as fixed effects. Post-hoc Fisher’s LSD tests were performed to check for differences between groups. Areas under the curve (AUCs) and incremental AUCs (iAUCs) were calculated using the trapezoid rule. For exploratory correlation analyses, Pearson’s correlation coefficients were calculated, using pairwise matching, and Partial correlations adjusting for covariates i.e., age and BMI, were additionally conducted. There was minimal missingness throughout both datasets, with data for all applicable variables missing completely at random (MCAR) per Little’s MCAR test. Outliers beyond the mean + 3 * SD range within each group were removed. A secondary analysis was performed after imputation using last observation carried forward, which was only applicable to a specific subset of variables with few missing values, and produced identical or overwhelmingly similar results (data not shown).

For the analysis and unsupervised hierarchical clustering of postprandial and hormonal data we implemented an approach using packages dplyr, tidyr, VIM, ggplot2 and reshape2 on R. We first performed an one-way analysis of variance (ANOVA) of all applicable postprandial variables (metabolites, lipids, NMR indices and hormones) to define variables that changed postprandially by group. After applying the Benjamini–Hochberg false discovery rate (BH-FDR) correction to account for multiple comparisons, significant features were averaged across datapoints, z-scored (scaled) and hierarchically clustered to create the heatmap. Intra-cluster features were further plotted across each patient group to display their time-related course across the MMT. For all intra-cluster elements, we ran mixed effects models with fixed effects time, group, and time * group in a manner identical to the one described above for the hormones, also applying the false discovery rate correction. For the 2-dimensional classifiers, PCA biplots were drawn using omics and applicable hormonal features after scaling. If any, missing values in clinical features were imputed according to column means. Plotted vectors were selected from the top loadings’ absolute length and plotted as overlaid arrows over the biplots. PLS-DA was performed on MetaboAnalystR using time * group as a sample grouping factor, which additionally extracted the top features and corresponding VIF values, reflecting the contribution of each variable to component separation.

### Ethics

Study 1 was approved by the Institutional and Ethics review boards of Laiko General Hospital, and Study 2 was approved by the Institutional and Ethics review board of Ippokration Hospital. Both studies received exempt status approval by the Beth Israel Deaconess Medical Center (BIDMC) Institutional Review Board (IRB), and conducted in accordance with the Declaration of Helsinki. All participants provided written informed consent. Samples were analyzed blindly at both BIDMC, Boston MA, USA and Ansh Laboratories, Webster, TX in the USA and data were statistically analyzed at BIDMC, Boston MA, USA.

All authors had access to the study data and reviewed and approved the final manuscript.

## Results

### Study 1

#### Anthropometric and basic biochemical variables

Baseline characteristics of participants with normal weight and obesity are presented in Table 1. There were expected between-groups differences in body composition and basic lipemic and glycemic indices, with leaner individuals displaying markedly lower BMI (22.1 ± 1.9 kg/m^2^ vs. 37.8 ± 5.4 kg/m^2^, *p* < 0.001), waist (75.8 ± 6.7 cm vs. 109.1 ± 12.5 cm, *p* < 0.001) and hip circumferences (95.2 ± 5.6 cm vs. 127 [121–134] cm, *p* < 0.001) compared with the group with obesity. Liver function tests were similar, with the exception of AST which attained significance, being higher in people with obesity when adjusted for BMI and sex (*p* = 0.025). Total cholesterol was similar between groups, whereas HDL-C was higher in people with normal weight compared with those with obesity and triglycerides were markedly elevated in people with obesity, differences which were primarily BMI- and sex-determined. Insulin, fasting glucose, HOMA-IR, and HbA1c were also higher in people with obesity, with no participants displaying prediabetes.

**Table 1:** Study 1 Baseline characteristics

	All (n = 30)	People with normal weight (n = 15)	People with obesity (n = 15)	*p*-value^a^	*p*-value^b^	*p*-value^c^
Age (years)	30.5(28–36)	29(27.5–33)	35.8 ± 8.7	0.092	0.07	NA
Sex (n = female)	21	11	10	0.715	NA	NA
*Anthropometric variables*						
Weight (kg)	87.5 ± 29.1	62.6 ± 11.2	112.3 ± 17.4	**< 0.001**	0.397	**< 0.001**
BMI (kg/m^2^)	27.6(21.8–37.5)	22.1 ± 1.9	37.8 ± 5.4	**< 0.001**	NA	**< 0.001**
Waist circumference (cm)	93.1 ± 19.7	75.8 ± 6.7	109.1 ± 12.5	**< 0.001**	0.387	**< 0.001**
Hip circumference (cm)	119(95–127)	95.2 ± 5.6	127(121–134)	**< 0.001**	0.6	**< 0.001**
Waist/Hip ratio	0.8 ± 0.1	0.8 ± 0	0.8 ± 0.1	**0.091**	0.563	0.2
*Liver function*						
AST (U/L)	16.5(14–18.8)	16.7 ± 2.8	17(14.5–18)	0.835	**0.025**	0.24
ALT (U/L)	15.5(12–21.5)	14(12–17.5)	18(14.5–26)	0.061	0.231	0.116
GGT (U/L)	15(11–23)	13(12–19.5)	19(11–28)	0.483	0.122	0.936
*Lipid profile*						
Total cholesterol (mg/dl)	185.5(161.2–206)	184(167.5–200.5)	184.1 ± 31.4	0.934	0.174	0.832
HDL-C (mg/dl)	57.3(45.5–72.5)	67.3 ± 14.2	45.8(43.5–55.3)	**0.005**	0.095	**0.014**
LDL-C (mg/dl)	109.7 ± 31.5	102.9 ± 32.9	117.1 ± 29.3	0.229	0.449	0.272
Triglycerides (mg/dl)	70(57–82.8)	62.1 ± 21.1	77(69–121)	**0.008**	0.8	**0.026**
*Glucose metabolism*						
Insulin (μIU/ml)	9.4(6.7–14.9)	6.8 ± 2.3	15.2(12.5–17.9)	**< 0.001**	0.861	**0.027**
Glucose (mg/dl)	85(78–89.2)	80.6 ± 7.4	88(84.5–90.8)	**0.05**	0.066	0.176
HOMA-IR	5.2 ± 0.2	5 ± 0.2	5.3(5.3–5.4)	**0.002**	0.308	**0.004**
HbA1c (%)	9.4(6.7–14.9)	6.8 ± 2.3	15.2(12.5–17.9)	**< 0.001**	0.861	**0.027**

Bold values indicate the statistically significant *p*-values

Data are presented as mean ± SD if normally distributed and as median with IQR if not normally distributed

*p*-value^a^: T-test comparing groups, reflecting the significance of an independent sample T-test for normally distributed variables or a Mann–Whitney U-test for non-normally distributed variables

*p*-value^b^: Analysis of covariance (ANCOVA) between individuals with normal weight versus individuals with obesity adjusted by BMI, and Sex

*p*-value^c^: Analysis of covariance (ANCOVA) between individuals with normal weight versus individuals with obesity adjusted by Waist hip, Sex, Age

#### The postprandial levels of total and H-specific GDF15 are similar between people with normal weight and obesity following consumption of a high-fat meal

**Fig. 1:**
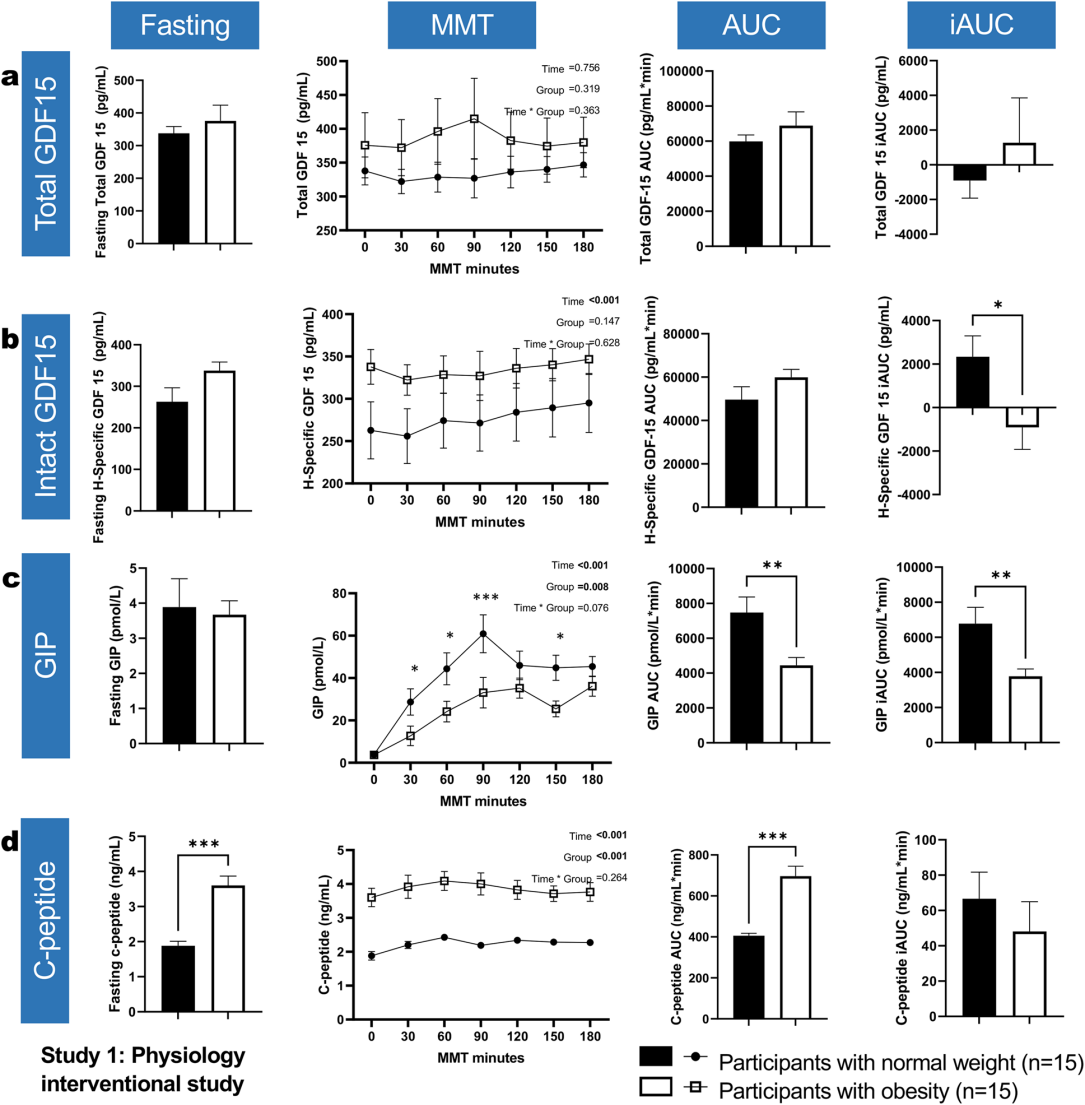
Fasting levels of total and H-specific GDF15, GIP, and C-peptide and their response to a mixed meal test in Study 1. Bars/points, and error bars reflect means ± SEM. Within bar charts, *, **, *** for the unpaired t-test with Welch’s correction comparing columns. Within line graphs, Time, Group and Time * Group for the fixed effects in the linear mixed effects models; and *, **, *** for the post-hoc LSD tests comparing groups within each respective timepoint

Fasting total and H-specific GDF15 were not significantly different between the two groups (*p* = 0.48 and *p* = 0.07 respectively) (Fig. 1a, b). Consumption of a meal with elevated caloric fat content resulted in no significant between-groups differences in either total or H-specific GDF15 AUC (*p* = 0.312 and 0.155, respectively). The iAUC of total GDF15 was likewise similar (*p* = 0.44), whereas H-specific GDF15 displayed a more accentuated time-related increasing trend in individuals with normal weight (*p*-time < 0.001) as indicated by a markedly higher iAUC (2336 ± 3720 vs. − 908 ± 3927 in people with normal weight vs. with obesity, respectively; *p* = 0.02) (Fig. 1).

#### GIP is robustly upregulated in people with normal weight whereas C-peptide remains higher in people with obesity, following consumption of a high-fat meal

Fasting levels of GIP were similar across the two groups (*p* = 0.813). Yet the postprandial secretion of GIP was markedly accentuated in the normal-weight group compared with people with obesity (*p*-treatment = 0.008), peaking at t = 120 min, in contrast with a delayed secretion and peak at t = 180 min in the obesity group. This was further reflected in the marked differences in postprandial AUC (7480 ± 3457 vs. 4444 ± 1747 pmol/L * min, in people with normal-weight and obesity, respectively; *p* = 0.006) and iAUC (6781 ± 3597 vs. 3783 ± 1596 likewise; *p* = 0.008) (Fig. 1c). On the contrary, fasting C-peptide was markedly upregulated in people with obesity (3.6 ± 1.04 ng/mL vs. 1.88 ± 0.49 ng/mL likewise; *p* < 0.001), potentially indicating a milieu of increased insulin resistance. There was a robust difference in the overall AUC of C-peptide, reflective of the baseline difference, being more pronounced in people with obesity (406 ± 49 vs. 696 ± 189, respectively; *p* < 0.001), whereas C-peptide iAUC was similar between groups (*p* = 0.42) (Fig. 1d).

### Study 2

#### Anthropometric and basic biochemical variables

Baseline anthropometric and biochemical of the MASLD and healthy controls (n = 13 and n = 14, respectively) are presented in Table 2. Twenty-seven individuals underwent an OGTT. The controls were more than a decade younger than the MASLD patients (p = 0.002). The mean BMI was 24.7 ± 2.5 kg/m^2^ for the controls, significantly different from that for MASLD patients (30.1 [29.4–31.4] kg/m^2^) (*p* < 0.001). In addition, MASLD patients had significantly larger waist and hip circumferences compared to the controls (*p* = 0.002 and *p* = 0.031, respectively). As expected, the MASLD group had the least favorable profile of liver function tests, with significantly higher levels of AST and ALT compared to the control group (*p* = 0.018 and *p* = 0.009, respectively) which were primarily BMI- and sex-determined. Lipids were similar between groups, whereas glycemic indices were less favorable in the MASLD group compared to the controls, with significantly higher insulin (*p* = 0.002), glucose levels (*p* = 0.006), HOMA-IR (*p* < 0.001) and HbA1c (*p* = 0.005) (Table 2).

**Table 2:** Study 2 baseline characteristics

Parameters	All (n = 27)	Controls (n = 14)	MASLD (n = 13)	*p*-value^a^	*p*-value^b^	*p*-value^c^
Age (years)	47.8 ± 13.8	38.5 (30.5–42.5)	55.6 ± 11	**0.002**	**0.002**	NA
Sex (n = female)	14	10	4	**0.042**	NA	NA
*Anthropometric variables*						
Weight (kg)	79.8 ± 18.2	68.3 ± 10	92.2 ± 17	**< 0.001**	0.661	**< 0.001**
BMI (kg/m^2^)	27(24.8–30.1)	24.7 ± 2.5	30.1(29.4–31.4)	**< 0.001**	NA	**< 0.001**
Waist circumference (cm)	98(93–102)	93 ± 7	102(98–122)	**0.002**	0.587	**0.041**
Hip circumference (cm)	106 ± 12	101 ± 11	111 ± 11	**0.031**	0.55	**0.043**
Waist/Hip ratio	1 ± 0.1	0.9(0.9–0.9)	1 ± 0.1	0.09	0.91	NA
*Liver function*						
AST (U/L)	28 ± 12	23 ± 10	33 ± 12	**0.018**	0.122	0.16
ALT (U/L)	27 ± 14	16(15–22)	34 ± 11	**0.009**	0.187	**0.003**
GGT (U/L)	17(13–23)	15(12–21)	19(15–26)	0.166	0.377	0.169
*Lipid profile*						
Total cholesterol (mg/dl)	174 ± 36	175 ± 24	172 ± 46	0.809	0.61	0.523
HDL-C (mg/dl)	48 ± 14	51 ± 10	42(36–46)	0.333	0.93	0.591
LDL-C (mg/dl)	106 ± 27	105 ± 17	108 ± 36	0.775	0.416	0.801
Triglycerides (mg/dl)	104 ± 46	98 ± 32	109 ± 57	0.547	0.682	0.95
*Glucose metabolism*						
Insulin (μIU/ml)	18.2 ± 8.1	13.9 ± 7.2	22.9 ± 6.4	**0.002**	0.218	**0.006**
Glucose (mg/dl)	95 ± 13	88 ± 8	102 ± 14	**0.006**	0.042	0.256
HOMA-IR	78.6 ± 38.6	56.1 ± 31.9	102.9 ± 29.9	**< 0.001**	0.088	**0.009**
HbA1c (%)	5.4(5–5.7)	5.1 ± 0.4	5.8 ± 0.7	**0.005**	0.121	0.173

Bold values indicate the statistically significant *p*-values

Data are presented as mean ± SD if normally distributed and as median with IQR if not normally distributed

*p*-value^a^: T-test comparing groups, reflecting the significance of an independent sample T-test for normally distributed variables or a Mann–Whitney U-test for non-normally distributed variables

*p*-value^b^: Analysis of covariance (ANCOVA) between healthy individuals versus individuals with MASLD adjusted by BMI, and Sex

*p*-value^c^: Analysis of covariance (ANCOVA) between healthy versus MASLD subjects adjusted by waist hip, Sex, Age

#### Fasting total GDF15 levels are markedly elevated in MASLD

**Fig. 2:**
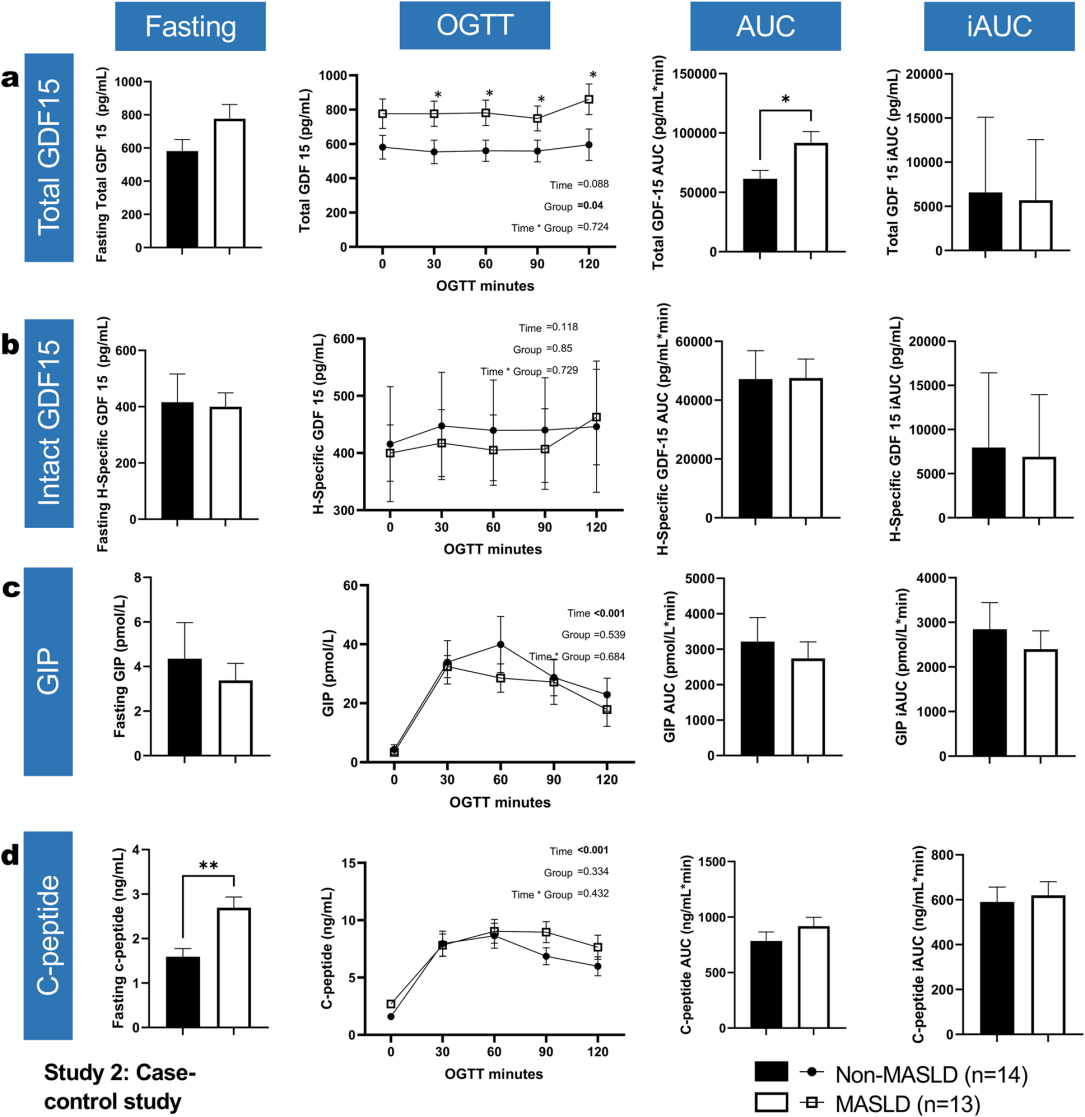
Fasting levels of total and H-specific GDF15, GIP, and C-peptide and their response to 75 g OGTT in Study 2. Bars/points, and error bars reflect means ± SEM. Within bar charts, *, **, *** for the unpaired t-test with Welch’s correction comparing columns. Within line graphs, Time, Group and Time * Group for the fixed effects in the linear mixed effects models; and *, **, *** for the post-hoc LSD tests comparing groups within each respective timepoint

Regarding baseline total and H-specific GDF15 levels, total GDF15 was similar between MASLD patients and controls (776.1 ± 296.4 vs. 581.5 ± 230.6 pg/mL, *p* = 0.09). H-specific GDF15 levels were likewise not significantly different between those groups (Fig. 2a, b). Fasting C-peptide levels were markedly different between MASLD patients compared with controls (1.59 vs. 2.69 ng/mL, *p* = 0.002) (Fig. 2d), while GIP levels likewise did not differ between the two groups (4.34 vs. 3.37 pmol/L, *p* = 0.58) (Fig. 2c).

#### Total and H-specific GDF15, C-peptide and GIP in response to 75-g OGTT

Postprandial GDF15 increased throughout the MMT in the MASLD group across all timepoints (group = 0.04, between-group post-hocs < 0.05). The AUC for total GDF15 levels was significantly higher in the MASLD group compared to the control group (61,399 ± 26,388 vs. 91,673 ± 34,508 pg/mL * min, *p* = 0.018) (Fig. 2a). H-specific GDF15 levels were not significantly different between people with MASLD and controls (47,155 ± 36,145 vs. 47,518 ± 23,308 pg/mL * min vs. *p* = 0.957) (Fig. 2b).

As for the AUCs for C-peptide and GIP levels, they did not differ between the two groups (*p* = 0.25 and *p* = 0.567, respectively) (Fig. 2c, d).

#### Association of GDF15, C-peptide and GIP with markers of the metabolomic/lipidomic profile in all individuals

**Fig. 3:**
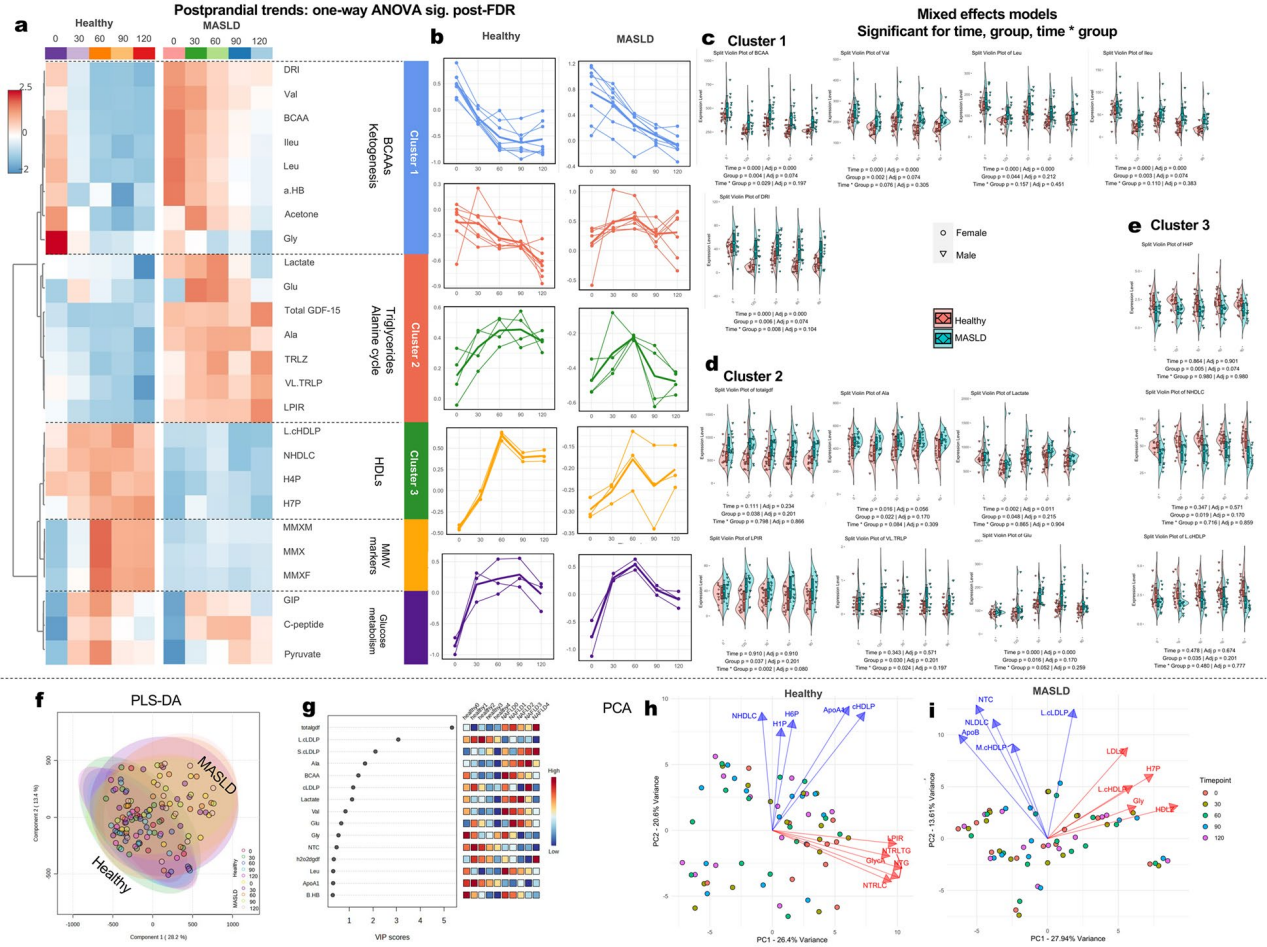
Fasting and postprandial metabolipidomic profiles, GDF15, C-peptide and GIP in Study 2. **a** Hierarchically clustered heatmap of top FDR-corrected metabolite and hormonal variances during the OGTT for both groups. Unsupervised clustering pinpointed 5 distinct clusters which contain functionally related and commonly regulated molecules across healthy and MASLD participants, with distinct time courses elucidated in (**b**), demonstrating the overall normalized trends of all features (simple lines) and the aggregate average line of each cluster (thicker line). **c**, **d**, **e** Split violin plots of the mixed effects models analysis with factors time (denoting OGTT minutes), group (denoting healthy vs. MASLD) and the time * group interaction, showing only molecules with either significant fixed effect and accompanied by FDR-adjusted corresponding *p*-values. These molecules also belong to the clusters shown in (**a**) and (**b**) and are presented likewise. Within-violin bars represent interquartile ranges, rhombuses means, and error bars standard deviations. Circles represent female and triangles male participants. **f** PLS-DA of all group-time combinations showcasing a distinct overlay and trend for differentiation between MASLD and healthy, alongside VIP scores of the top significant variables driving this differentiation (**g**). **h** and **i** unsupervised PCA within groups analyses of the circulating metabolipidome during all OGTT timepoints demonstrating vectors of the top 5 significant variables driving OGTT variance across each component, for healthy participants and participants with MASLD, respectively

To further explore any potential pathophysiological role of GDF15, C-peptide, and GIP in metabolomic/lipidomic pathways, we clustered and plotted the postprandial levels of all applicable metabolites, lipids, and features from the 60-variable LabCorp platform, alongside postprandial levels of examined hormones. The fasting and postprandial profiles are presented in Fig. 3. The top variables (omics and hormones) according to ANOVA during the MMT, following the BH-FDR correction, were hierarchically clustered into 5 clusters that pinpointed functionally related and commonly regulated molecules (Fig. 3a, b). Cluster 1 (blue) contained BCAAs and ketogenesis-related molecules and displayed a downward trend until the end of the OGTT for both groups, which was notably steeper in healthy participants compared with MASLD. Cluster 2 (red) contained triglyceride and components of the alanine cycle, alongside GDF15, and was downregulated in healthy participants while remaining relatively stable for MASLD patients. Cluster 3 (green) contained HDLs and was stable in MASLD patients during the OGTT, all the while increasing in healthy participants. MMV markers and glucose metabolism clusters showed an increase until the end of the OGTT for both groups (Fig. 3b). Further examination of omics variables with mixed effects models akin to the ones ran for the hormones (Figs. 1, 2) revealed BCAAs, (valine, leucine, isoleucine), the NMR-based Diabetes Risk Index (DRI) as well as Lactate and glutamine (Fig. 3c–e).

To fully map data dimensionality and pinpoint the postprandial features separating healthy from MASLD status during the MMT, we performed a partial least squares discriminant analysis on postprandial omics and hormonal data, clustering according to healthy versus MASLD status (Fig. 3f). There was a clear differentiation of the MASLD biochemical profile regardless of meal timepoint, with the top significant variables responsible for this differentiation per variance importance projection scores (VIP) being total GDF15, large LDL particles (L.cLDLP), small LDL particles (S.cLDLP), alanine (Ala), and total BCAA (Fig. 3g). We also present unsupervised principal component analyses of the postprandial omics and hormonal profiles indicating the most significant variables driving OGTT variance across each component for each group, notably indicating HDLs and ApoA1 as significant features in the healthy group, and ApoB, LDL particles and glycine as significant features in the MASLD group (Fig. 3h, i).

To further assess potential relationships of co-regulation between omics variables and hormones, we performed exploratory correlations (Suppl. Tables 2–4) of total and H-specific GDF15, C-peptide, and GIP with 60 lipids and metabolites (Suppl. Table 1) at baseline (fasting) and during the OGTT (AUCs and iAUCs) in Study 2. Our analysis showed a negative relationship between both baseline and AUC total GDF15 with the large LDL particles (L-LDLP), mean LDL size (LDLZ), LDL cholesterol (LDLC), glycine, and the proinflammatory marker GlycA (Suppl. Tables 2, 3). To the contrary, a positive relationship between baseline and AUC total GDF15 and the metabolic malnutrition index (MMX) was observed (Suppl. Tables 2, 3). However, none of these correlations persisted after adjusting for age and BMI (Suppl. Tables 2, 3). The AUC of total GDF15 was positively correlated with large and very large triglyceride-rich lipoprotein particles, the triglyceride size (TRLZ), isoleucine, alanine, and glutamine, ketone bodies, acetone, and the Lipoprotein Insulin Resistance (LP-IR) Score and Diabetes Risk Index (DRI). Of note, the LP-IR Score is a lipoprotein particle-derived score [48] and the DRI was developed by combining the LP-IR Score and the branched chain amino acids (BCAAs), valine (Val) and leucine (Leu) [49]. Both of them have been associated with increased risk of T2D. The AUC of total GDF15 was further negatively correlated with large LDL particles, LDL cholesterol, and H7P HDL cholesterol. These correlations did not persist after adjustments (Suppl. Table 3). However, the iAUC of total GDF15 was positively correlated with a plethora of lipid subspecies and BCAAS, with H-specific GDF15 also being correlated with lipids, correlations which remained significant even after adjustments. (Suppl. Table 4). The significance of all correlation coefficients was however lost upon applying corrections for multiple comparisons.

## Discussion

Our study uncovers novel data on the physiology of GDF15, GIP, and C-peptide in leanness, obesity, and MASLD. In study 1, we show that both fasting and postprandial GDF15 levels are unaffected by weight status in healthy individuals, whereas the levels of GIP are markedly elevated in leaner individuals compared with people with obesity and are robustly increased following a high-fat meal. To the contrary, the baseline C-peptide levels and their overall AUC were markedly elevated in people with obesity compared with lean subjects, without any significant difference between the two groups after a fatty meal. Expanding upon the pathophysiology of MASLD through study 2, we further show that MASLD is characterized by higher total but not H-specific GDF15 levels following an OGTT, whereas GIP and C-peptide remain unaffected. We additionally report promising exploratory associations of GDF15 levels with metabolomic and lipidomic variables, indicating negative correlations with LDL particles and the proinflammatory marker GlycA.

The role of GDF15 in obesity remains obscure and is possibly different between humans and animals. GDF15 knockout mice consuming a high-fat diet have worsened glucose tolerance and metabolic rate, indicating a possible protective role of GDF15 in obesity [24], whereas a microarray study has shown that total GDF15 is upregulated in obese mice, rats, and humans [50]. A comprehensive physiology study recently evaluated the postprandial perturbations of GDF15 following glucose or meal loads, caloric restriction, or hypercaloric loads. Specifically, it was shown that GDF15 levels remain unchanged following oral glucose or meal ingestion. A sustained caloric deficit produces marked increases in GDF15 in humans, but not mice, whereas short-term (7 days) hypercaloric loads do not affect GDF15 levels in mice or humans, and longer-term hypercaloric loads (4 weeks) increase GDF15 levels in mice [17]. Herein, both during fasting and following the ingestion of a high-fat meal over 3 h, we show that GDF15 is not significantly different between people with distinct weight status (leanness vs. obesity). It is unclear whether these observations are influenced by underlying metabolic stress and inflammation, which are characteristic of obesity [51], given the nature of GDF15 as a mitokine [52]. However, these outcomes have fueled the development of GDF15 agonists against metabolic disease. YH34160, a novel GDF15 fusion protein, has been shown to effectively reduce body weight, both alone and when used in combination with a glucagon-like peptide 1 (GLP-1) agonist, in diet-induced obese mice [53]. On the other end of the spectrum and in line with our observations, a cross-sectional physiology interventional study comparing operated individuals who had undergone bariatric surgery with unoperated otherwise healthy controls, consuming either liquid carbohydrates, lipids, or a liquid mixed meal, reported that fasting and postprandial GDF15 were not increased or affected by nutrient type and surgery [54].

As we mentioned above, in MASLD, GDF15 has been studied in animals and humans [30, 31]. In mice, it has been suggested that an observed increase of GDF15 levels acts as a compensatory mechanism to prevent MASLD progression [16]. GDF15-knockout mice develop severe hepatic inflammation and fibrosis, while treatment with recombinant GDF15 seems to alleviate inflammation and fibrosis [15, 16]. In humans, GDF15 has been found to be associated with MASLD [15] and GDF15 levels represent an independent determinant of fibrosis severity in MASLD; the more severe the chronic liver disease is, the higher the GDF15 levels are, apparently acting as a compensatory mechanism [30]. However, limitations and controversial results similar to those in obesity have been reported. These conflicting results could be explained, in part, by the existence of GDF15 polymorphisms, i.e. rs1058587, which changes the 202 residue from histidine (H) to aspartic acid (D), and interferes with GDF15 measurements and reported levels [55] although it is not clear whether the variant alters the bioactivity of the molecule [28, 56, 57]. Recently, the possibility to accurately measure the total form of the molecule (irrespectively of the polymorphisms) and the H-specific part of it (without measuring the molecules with polymorphisms) has emerged as a solution to this problem [28, 29].

In this context, higher concentrations of total GDF15 seem to exist in proinflammatory milieus. A recent study in youth with overweight/obesity reported, without discriminating between total and H-specific GDF15, that GDF15 concentrations varied with alterations in liver fat content [31]. Another study using a biopsy-proven MASLD cohort of 190 subjects showed that GDF15 levels are significantly associated with risk of advanced fibrosis even after adjustment for age, sex, smoking habit, and metabolic factors (odds ratio [OR], 4.27; 95% confidence interval [CI] 1.04–17.63), but not with MASH risk [30]. Also, GDF15 concentrations were elevated with increased lipid synthesis in the liver of a mouse model [18]. Our findings are consistent with these previous studies and advance them by reporting both total and H-specific GDF15 at baseline and during OGTT. It can be hypothesized that GDF15 may protect against the inflammatory response in fatty liver disease [16]. Besides, overexpression of GDF15 may attenuate hepatic inflammation and fibrosis [16]. Of note, in hepatocytes, GDF15 expression is considered to be promoted by interleukin (IL)-1β signaling and ER stress which are involved in MASLD development and progression [58, 59]. Thus, GDF15 coordinates tolerance to inflammatory injury, and may be involved in the regulation of lipid metabolism [18]. GDF15 has been shown to be necessary for surviving both bacterial and viral infections, as well as sepsis, since it was needed for hepatic sympathetic outflow and triglyceride availability control [18]. In addition, it has been suggested that GDF15 can dramatically upregulate hepatic fatty acid oxidation and ketogenesis [60, 61].

Circulating levels of total and H-specific GDF15 displayed minor and non-significant variation during an OGTT and reverted to the initial levels by the end of the procedure. This finding is consistent with what has previously been shown regarding total GDF15 in overweight or subjects with obesity [62–64]. Although Patel et al. did not observe any significant change in total GDF15 levels during OGTT, they reported a fall in GDF15 levels an hour after the consumption of a mixed meal [17]. High glucose levels promote GDF15 expression in human endothelial cells [65] and its levels increase during euglycemic hyperinsulinemic clamps [66]. Subsequently, it has been suggested that glucose and insulin are needed for the GDF15 transcription and secretion in the human hepatic cell line HepG2. Despite the above, total and H-specific GDF15 levels do not appear to be affected, even after glucose load during OGTTs. The total GDF15 responses to OGTT differ significantly between healthy and MASLD patients, thus indicating that GDF15 response to a glucose load is relatively dampened in healthy subjects. That has also been observed in another study, which did not discriminate between total and H-specific GDF15, in lean and otherwise healthy overweight subjects [64].

GIP, an incretin promoter of postprandial insulin secretion alongside GLP-1 has been frequently implicated in glucose homeostasis [67, 68] and is being leveraged for the treatment of metabolic disease [69]. GIP has also been suggested to possess an obesogenic role in both animal [70] and human models [71]. In humans, early studies showed that in people with obesity, GIP is more accentuated in response to oral fat compared with glucose, whereas in normal-weight individuals, co-ingestion of fat and glucose produced a less pronounced GIP released compared to fat alone, further suggesting that insulin, which is not released when consuming fat alone, inhibits GIP release [72]. A pioneering study investigating GIP-receptor deficient mice showed that weight and adiposity were physiologically influenced under normocaloric feeding; however, a high-fat diet attenuated body weight and fat mass gain, and preserved normal insulin sensitivity [73].

Although an exaggerated C-peptide response is noted in insulin resistant subjects, as MASLD patients are, compared with the more insulin sensitive group [74], we did not observe a significant difference between the two studied groups. Likewise, there was no difference in GIP. However, in humans, circulating levels of GIP have been shown to be inversely correlated with insulin sensitivity and thus, the postprandial release of GIP has been linked to the presence of MASLD [36]. Interestingly, fasting GIP plasma levels did not differ between biopsy proven MASH patients and control healthy subjects in a HepatoMetabolic Clinic in Italy. Moreover, it has been suggested that, after OGTT, GIP concentrations do not differ significantly between MASLD and healthy individuals [75].

It was also explored whether total and H-specific GDF15, C-peptide, and GIP levels either at baseline or during an OGTT (AUC) are associated with any metabolomic/lipidomic pathways. Towards this direction a multi-omics approach was followed and it was checked whether GDF15, C-peptide, and GIP levels correlate with any of the metabolites or lipids in all individuals (subjects with MASLD and controls). Total GDF15 presented a moderately strong negative correlation with LDLZ (the LDL size) and LDLC (the measurement of the cholesterol concentration of the LDL particles). LDL cholesterol is considered as a major risk factor for development of myocardial infarction (MI) and atherosclerotic cardiovascular disease [76]. Also, several studies have suggested that a high concentration of small and dense LDL particles is related to an increased risk for coronary heart disease [77]. On the contrary, GDF15 seems to exert a cardioprotective role [78] and, probably, for this reason the total and H-specific GDF15 levels during the OGTTs were positively correlated with various lipid subspecies; a finding similar to that of our previous study [28]. Plasma cholesterol levels of high-density lipoprotein (HDL) have also been associated with cardioprotection [79]. It has also been suggested that specific HDL particles may be more important in predicting or preventing cardiovascular disease [80]. H7P HDL cholesterol is negatively correlated with the AUC of total GDF15 H-specific, but this correlation was not still significant after adjustments for BMI and age. To this context, a negative correlation between GDF15 and H6P and H7P was recently reported [28].

Alanine along with lactic acid are the traditional hallmarks of defective mitochondrial oxidized phosphorylation (OXPHOS) which has been linked to several cardiovascular diseases [81, 82]. Herein we show a positive correlation between alanine and the AUC of total GDF15. In agreement with this finding, GDF15 is recognized also as a biomarker of endothelial dysfunction, atherosclerosis, and heart failure [78, 83]. Additionally, it appears that GDF15 is induced in the cells by hypoxia and this effect is related in various cells and tissues. Interestingly, GDF15 expression is highly induced in cardiomyocytes after ischemia/reperfusion and in the heart within hours after MI [84]. In accordance with the prior findings is the moderately strong positive correlation between GDF15 and age. Several studies have confirmed the increase of GDF15 levels by ageing and in response to cellular stress and mitochondrial dysfunction [78]. These data and underlying mechanisms need to be studied further.

Perturbations in numerous metabolic and biological pathways in response to a glucose challenge have been demonstrated. Suppression of ketogenesis, proteolysis (ileu), and increased glycolysis (lactate) are regulated via insulin actions and these are attenuated in the insulin-resistant state [85], as MASLD is supposed to be and as we demonstrated using metabolipidomic profiles and unsupervised clustering of functionally related and commonly regulated molecules across healthy and MASLD participants in Study 2. A blunted response in all key axes, proteolysis, lipolysis, ketogenesis, and glycolysis, is observed in insulin resistant states during the OGTT [86], as it was also observed in our study. Similarly, BCAA levels have been shown to decrease more in lean compared to obese subjects [87] and increased concentrations of BCAAs and their metabolites have been found in MASLD and MASH patients [88–90]. Interestingly, a dysregulation of BCAAs metabolism (val, leu, ileu) has been found also in obese adolescents with MASLD performing OGTT as well [91].

We present herein, for the first time the measurements and comparisons of total and H-specific GDF15 levels between healthy adults and MASLD patients as well as their changes during OGTT. Importantly, H-specific has not been studied previously in these groups of subjects and this is one of the novel aspects of the study. Our metabolomic/lipidomic analysis could be considered as an all-embracing and unbiased approach that can motivate further research on GDF15, for which little is known. Additional metabolites, lipid subgroups and even proteomics analysis and mechanistic studies can gain ground in the future. However, we are aware that our study has some limitations, mainly because there are no hepatic histological data available, and due to the small sample size, which was however sufficient to demonstrate statistically significant differences in GDF15 levels between the studied groups. For these reasons, our results should be confirmed in further larger longitudinal studies implementing histological confirmation of MASLD severity.

## Conclusions

In conclusion, both fasting and postprandial GDF15 levels are independent of weight status in otherwise healthy individuals. GIP is markedly higher in leaner individuals and is upregulated after a high-fat meal, while C-peptide and its overall AUC after a high fat meal ingestion is markedly elevated in people with obesity compared with lean subjects. For the first time it is shown that total serum GDF15 levels are significantly increased in MASLD patients during OGTT and remain significantly higher compared to otherwise healthy individuals, pointing to a role for GDF15 as a mitokine with important roles in the pathophysiology and possibly therapeutics of MASLD. Consequently, GDF15 may be identified as a potential biomarker for diagnosing MASLD. Future larger, prospective studies are needed to confirm the role of GDF15 in the pathophysiology of MASLD and reveal the potential metabolic links between GDF15 and MASLD in adults.

### Supplementary Information

Supplementary Material 1.

## Data Availability

The datasets used and analyzed during the current study are available from the corresponding author on reasonable request. The full trial protocol can be accessed at clinicaltrials.gov.

## Abbreviations

ALT: Alanine transaminase
AST: Aspartate aminotransferase
AUC: Area under the curve
BIDMC: Beth Israel Deaconess Medical Center
BMI: Body mass index
CKD: Chronic kidney disease
ELISA: Enzyme-linked immunosorbent assay
ER: Endoplasmic reticulum
FLI: Fatty liver index
GDF15: Growth differentiation factor 15
GGT: Gamma-glutamyl transferase
GIP: Glucose-dependent insulinotropic polypeptide
GLP-1: Glucagon-like peptide 1
HDL-C: High-density lipoprotein cholesterol
iAUC: Incremental AUC
IRB: Institutional Review Board
LDL-C: Low-density lipoprotein cholesterol
MCAR: Missing completely at random
MMT: Mixed-meal test
MMX: Metabolic malnutrition index
MASLD: Metabolic dysfunction-associated steatotic liver disease
MASH: Metabolic dysfunction-associated steatohepatitis
NMR: Nuclear magnetic resonance spectroscopy
OGTT: Oral glucose tolerance test
TGFβ: ΤRansforming growth factor beta
