# Supplementary material for: Circulating total and H-specific GDF15 levels are elevated in subjects with MASLD but not in hyperlipidemic but otherwise metabolically healthy subjects with obesity
Source: Cardiovasc Diabetol. 2024 May 18;23:174. doi: 10.1186/s12933-024-02264-5 (PMC11102634; doi:10.1186/s12933-024-02264-5)
Supplement: Supplementary file 1 — Supplementary Material 1. [file 12933_2024_2264_MOESM1_ESM.docx]

**Supplementary Table 1:** -Omics abbreviation list.

| **Abbreviation** | **Analyte Description** | **Units** |
| --- | --- | --- |
| TRLP | Triglyceride rich lipoprotein (TRL) particles  (Total chylomicron & VLDL particles) | nmol/L |
| VL_TRLP | Very large TRL particles | nmol/L |
| L_TRLP | Large TRL particles | nmol/L |
| M_TRLP | Medium TRL particles | nmol/L |
| S_TRLP | Small TRL particles | nmol/L |
| VS_TRLP | Very small TRL particles | nmol/L |
| LDLP | Total low density lipoprotein (LDL) particles | nmol/L |
| L_LDLP | Large LDL particles | nmol/L |
| M_LDLP | Medium LDL particles | nmol/L |
| S_LDLP | Small LDL particles | nmol/L |
| HDLP | Total high density lipoprotein (HDL) particles | µmol/L |
| L_HDLP | Large HDL particles | µmol/L |
| M_HDLP | Medium HDL particles | µmol/L |
| S_HDLP | Small HDL particles | µmol/L |
| H7P | H7 subspecies of HDL | µmol/L |
| H6P | H6 subspecies of HDL | µmol/L |
| H5P | H5 subspecies of HDL | µmol/L |
| H4P | H4 subspecies of HDL | µmol/L |
| H3P | H3 subspecies of HDL | µmol/L |
| H2P | H2 subspecies of HDL | µmol/L |
| H1P | H1 subspecies of HDL | µmol/L |
| TRLZ | Mean TRL size | nm |
| LDLZ | Mean LDL size | nm |
| HDLZ | Mean HDL size | nm |
| TG | Total TRL | mg/dL |
| TC | Total Cholesterol | mg/dL |
| TRLTG | TRL triglycerides | mg/dL |
| TRLC | TRL Cholesterol | mg/dL |
| LDLC | LDL Cholesterol | mg/dL |
| HDLC | HDL Cholesterol | mg/dL |
| ApoB | Apolipoprotein B | mg/dL |
| ApoAI | Apolipoprotein A-I | mg/dL |
| BCAA | Total branched chain amino acids | µmol/L |
| Val | Valine | µmol/L |
| Leu | Leucine | µmol/L |
| Ileu | Isoleucine | µmol/L |
| Ala | Alanine | µmol/L |
| Mg | Magnesium | µmol/L |
| Ctr | Citrate | mg/dL |
| KetBod | Total ketone bodies | µmol/L |
| B-HB | β-hydroxybutyrate | µmol/L |
| a-HB | α-hydroxybutyrate | µmol/L |
| AcAc | Acetoacetate | µmol/L |
| Acetone | Acetone | µmol/L |
| Glu | Glucose | mg/dL |
| GlycA | GlycA | µmol/L |
| TMAO | Trimethylamine N-oxide | µmol/L |
| Betaine | Betaine | µmol/L |
| Choline | Choline | µmol/L |
| Lactate | Lactate | µmol/L |
| Pyruvate | Pyruvate | µmol/L |
| Glycine | Glycine | µmol/L |
| IVX | Inflammation Vulnerability Index | Score |
| MMX | Metabolic Malnutrition Index | Score |
| MVX | Metabolic Vulnerability Index | Score |
| Creatine | Creatine | mg/L |
| Creatinine | Creatinine | mg/dL |
| eGFR | Estimated glomerular filtration rate, CKD EPI 2021 | ml/min/1.73m^2^ |
| LP-IR | Lipoprotein Insulin Resistance Index | Score |
| DRI | Diabetes Risk Index | Score |

**Supplementary Table 2:** Spearman Correlation matrix of Fasting C-peptide, GDF15 (total and h-specific) and GIP with fasting metabolipidomic variables; unadjusted and adjusted for age and BMI.

|  | Fasting C-peptide | Fasting  C-peptide adjusted | Fasting Total GDF15 | Fasting Total  GDF15 adjusted | Fasting h-specific GDF15 | Fasting h-specific  GDF15 adjusted | Fasting GIP | Fasting GIP adjusted |
| --- | --- | --- | --- | --- | --- | --- | --- | --- |
| TRLP_0 | -0.38 | -0.02 | -0.09 | 0.00 | 0.01 | -0.27 | -0.23 | 1.00 |
| VL-TRLP_0 | 0.00 | 0.22 | 0.25 | 0.04 | 0.31 | -0.13 | 0.01 | 0.27 |
| L-TRLP_0 | -0.21 | 0.17 | 0.09 | 0.41 | 0.38 | 0.26 | 0.20 | 0.40 |
| M-TRLP_0 | -0.21 | 0.12 | 0.06 | 0.03 | -0.05 | -0.17 | -0.09 | **.57**^**^ |
| S-TRLP_0 | -0.37 | -0.19 | -0.32 | -0.11 | -0.18 | 0.28 | 0.14 | **.57**^**^ |
| VS-TRLP_0 | -0.17 | 0.06 | -0.01 | 0.04 | -0.02 | -0.46 | -0.36 | **.90**^**^ |
| cLDLP_0 | -0.12 | -0.42 | -0.39 | 0.11 | 0.17 | 0.24 | 0.15 | 0.36 |
| L-cLDLP_0 | -0.02 | **-.47**^*^ | **-.52**^*^ | 0.03 | -0.12 | 0.45 | 0.10 | 0.19 |
| M-cLDLP_0 | -0.28 | 0.12 | 0.11 | 0.16 | 0.17 | -0.18 | -0.12 | 0.30 |
| S-cLDLP_0 | 0.30 | -0.11 | 0.10 | -0.02 | 0.18 | -0.05 | 0.30 | 0.02 |
| cHDLP_0 | -0.27 | 0.06 | -0.10 | 0.23 | -0.08 | -0.32 | -0.40 | **.53**^*^ |
| L-cHDLP_0 | -0.01 | 0.05 | 0.08 | -0.27 | -0.10 | -0.19 | -0.10 | -0.37 |
| M-cHDLP_0 | -0.21 | -0.12 | -0.33 | 0.29 | 0.09 | 0.04 | -0.16 | 0.30 |
| S-cHDLP_0 | -0.13 | 0.11 | -0.01 | 0.22 | 0.02 | -0.36 | -0.42 | **.59**^**^ |
| H7P_0 | 0.17 | 0.00 | 0.05 | -0.06 | 0.03 | 0.33 | 0.35 | **-.58**^**^ |
| H6P_0 | -0.28 | 0.06 | -0.19 | -0.32 | -0.22 | -0.31 | **-.49**^*^ | 0.00 |
| H5P_0 | **.45**^*^ | 0.00 | 0.25 | 0.18 | 0.00 | -0.29 | -0.15 | 0.02 |
| H4P_0 | -0.36 | 0.10 | -0.29 | -0.08 | -0.33 | 0.44 | 0.09 | -0.22 |
| H3P_0 | 0.05 | -0.13 | -0.10 | 0.32 | 0.32 | -0.17 | -0.19 | 0.41 |
| H2P_0 | -0.16 | -0.01 | -0.17 | 0.21 | -0.03 | -0.30 | -0.43 | **.60**^**^ |
| H1P_0 | 0.14 | 0.32 | 0.29 | 0.09 | 0.17 | -0.27 | -0.09 | 0.09 |
| TRLZ_0 | 0.04 | 0.42 | 0.31 | 0.35 | 0.34 | -0.07 | -0.03 | 0.19 |
| LDLZ_0 | -0.11 | -0.43 | -0.41 | -0.05 | -0.17 | 0.36 | -0.07 | -0.03 |
| HDLZ_0 | 0.05 | -0.02 | 0.04 | -0.25 | -0.07 | 0.29 | 0.29 | **-.66**^**^ |
| NTG_0 | -0.30 | 0.07 | -0.04 | 0.22 | 0.24 | 0.05 | 0.07 | **.72**^**^ |
| NTC_0 | -0.23 | -0.34 | -0.37 | 0.11 | 0.05 | 0.20 | 0.08 | **.58**^**^ |
| NTRLTG_0 | -0.32 | 0.13 | 0.02 | 0.23 | 0.23 | 0.03 | 0.05 | **.73**^**^ |
| NTRLC_0 | -0.38 | 0.00 | -0.01 | 0.05 | -0.01 | -0.10 | -0.04 | **.96**^**^ |
| NLDLC_0 | -0.10 | **-.48**^*^ | **-.48**^*^ | 0.11 | 0.10 | 0.36 | 0.20 | 0.35 |
| NHDLC_0 | -0.22 | 0.09 | -0.09 | 0.03 | -0.02 | -0.11 | -0.18 | 0.02 |
| ApoB_0 | -0.13 | -0.38 | -0.39 | 0.10 | 0.14 | 0.19 | 0.11 | **.59**^**^ |
| ApoA1_0 | -0.39 | 0.03 | -0.16 | 0.12 | -0.15 | -0.30 | -0.34 | 0.39 |
| BCAA_0 | 0.06 | -0.04 | 0.08 | 0.24 | 0.24 | 0.08 | 0.13 | **.60**^**^ |
| Val_0 | 0.12 | -0.14 | -0.01 | 0.01 | 0.04 | 0.24 | 0.22 | **.54**^*^ |
| Leu_0 | -0.15 | -0.04 | -0.01 | 0.35 | 0.25 | -0.03 | -0.16 | **.50**^*^ |
| Ileu_0 | 0.11 | 0.19 | 0.40 | 0.30 | 0.33 | -0.03 | 0.12 | **.51**^*^ |
| Ala_0 | 0.43 | -0.17 | 0.23 | 0.44 | **.45**^*^ | -0.21 | -0.10 | 0.34 |
| Glu_0 | 0.12 | -0.24 | 0.18 | -0.10 | -0.15 | -0.02 | 0.12 | 0.42 |
| Ctr_0 | -0.03 | 0.38 | 0.09 | 0.27 | 0.13 | 0.40 | 0.11 | -0.15 |
| KetBod_0 | -0.16 | **.53**^*^ | 0.07 | -0.01 | **-.45**^*^ | 0.24 | 0.23 | -0.22 |
| B-HB_0 | -0.21 | **.51**^*^ | 0.15 | 0.02 | -0.39 | 0.24 | 0.28 | -0.25 |
| AcAc_0 | -0.08 | 0.03 | -0.21 | -0.25 | -0.36 | -0.04 | -0.18 | 0.27 |
| Acetone_0 | -0.14 | 0.36 | 0.02 | -0.18 | **-.53**^*^ | 0.01 | -0.13 | -0.01 |
| LPIR_0 | -0.06 | 0.37 | 0.29 | 0.33 | 0.28 | -0.02 | 0.05 | 0.36 |
| DRI_0 | -0.02 | 0.10 | 0.15 | 0.35 | 0.31 | 0.04 | -0.06 | **.57**^**^ |
| GlycA_0 | -0.05 | **-.50**^*^ | **-.60**^**^ | 0.27 | 0.07 | -0.15 | -0.20 | 0.30 |
| a-HB_0 | -0.07 | 0.06 | -0.15 | 0.10 | -0.03 | 0.40 | 0.16 | 0.31 |
| Gly_0 | 0.08 | **-.54**^*^ | **-.47**^*^ | 0.30 | 0.31 | -0.24 | -0.23 | -0.04 |
| Lactate_0 | **.54**^*^ | 0.15 | 0.25 | 0.29 | 0.41 | -0.12 | 0.01 | 0.06 |
| Pyruvate_0 | 0.39 | -0.19 | -0.17 | 0.04 | -0.18 | -0.04 | -0.25 | -0.15 |
| IVX_0 | 0.04 | -0.16 | -0.20 | -0.20 | -0.05 | 0.39 | 0.34 | **-.56**^**^ |
| IVF_0 | 0.03 | -0.15 | -0.19 | -0.23 | -0.11 | 0.40 | 0.35 | **-.57**^**^ |
| IVM_0 | 0.02 | -0.18 | -0.25 | -0.17 | -0.04 | 0.38 | 0.35 | **-.55**^*^ |
| MMX_0 | 0.19 | **.67**^**^ | **.60**^**^ | 0.05 | -0.11 | 0.21 | 0.22 | -0.41 |
| MMXF_0 | -0.06 | **.61**^**^ | 0.39 | 0.23 | 0.03 | 0.25 | 0.13 | -0.41 |
| MMXM_0 | 0.32 | **.54**^*^ | **.62**^**^ | -0.10 | -0.19 | 0.11 | 0.19 | -0.34 |
| MVX_0 | 0.13 | 0.07 | 0.09 | -0.16 | -0.13 | 0.42 | **.47**^*^ | **-.60**^**^ |
| MVXF_0 | 0.03 | 0.12 | 0.09 | -0.11 | -0.09 | **.49**^*^ | **.52**^*^ | **-.65**^**^ |
| MVXM_0 | 0.24 | 0.06 | 0.04 | -0.16 | -0.12 | 0.38 | 0.43 | **-.57**^**^ |

**. Correlation is significant at the 0.01 level (2-tailed)

*. Correlation is significant at the 0.05 level (2-tailed)

**Supplementary Table 3:** Spearman Correlation matrix of C-peptide, GDF15 (total and h-specific) and GIP postprandial AUCs with the postprandial AUCs of metabolipidomic variables; unadjusted and adjusted for age and BMI.

|  | C-peptide AUC | C-peptide AUC adjusted | Total GDF15 AUC | Total GDF15 AUC adjusted | H-specific GDF15 AUC | H-specific GDF15 AUC adjusted | GIP AUC | GIP AUC adjusted |
| --- | --- | --- | --- | --- | --- | --- | --- | --- |
| TRLP_AUC | -0.08 | -0.21 | 0.21 | 0.12 | 0.18 | 0.09 | -0.35 | -0.39 |
| VL-TRLP_AUC | 0.04 | -0.23 | **.48**^*^ | 0.25 | 0.20 | 0.10 | -0.21 | -0.29 |
| L-TRLP_AUC | 0.07 | 0.05 | **.48**^*^ | 0.37 | **.48**^*^ | **.49**^*^ | -0.09 | -0.16 |
| M-TRLP_AUC | -0.02 | -0.08 | 0.38 | 0.29 | 0.31 | 0.37 | -0.25 | -0.26 |
| S-TRLP_AUC | -0.20 | -0.11 | -0.16 | -0.18 | -0.22 | -0.14 | -0.28 | -0.30 |
| VS-TRLP_AUC | 0.04 | -0.21 | 0.28 | 0.20 | 0.32 | 0.13 | -0.30 | -0.32 |
| cLDLP_AUC | -0.22 | -0.07 | -0.23 | -0.27 | -0.14 | -0.09 | -0.37 | -0.34 |
| L-cLDLP_AUC | 0.01 | 0.25 | **-.53**^**^ | **-.48**^*^ | -0.33 | -0.24 | -0.17 | -0.21 |
| M-cLDLP_AUC | -0.12 | -0.23 | 0.22 | 0.09 | 0.19 | 0.08 | 0.07 | 0.10 |
| S-cLDLP_AUC | -0.05 | -0.24 | 0.31 | 0.12 | 0.16 | 0.10 | -0.29 | -0.25 |
| cHDLP_AUC | -0.08 | -0.15 | 0.34 | 0.40 | 0.21 | 0.37 | -0.16 | -0.12 |
| L-cHDLP_AUC | -0.02 | -0.05 | -0.31 | 0.03 | -0.30 | -0.36 | -0.12 | -0.16 |
| M-cHDLP_AUC | 0.08 | 0.13 | 0.12 | 0.24 | 0.06 | 0.26 | **-.42**^*^ | -0.39 |
| S-cHDLP_AUC | -0.12 | -0.22 | **.46**^*^ | 0.38 | 0.34 | **.41**^*^ | 0.03 | 0.03 |
| H7P_AUC | 0.11 | 0.04 | **-.43**^*^ | -0.36 | -0.37 | -0.35 | -0.02 | -0.06 |
| H6P_AUC | -0.13 | -0.16 | 0.04 | 0.37 | 0.13 | 0.02 | -0.22 | -0.19 |
| H5P_AUC | 0.10 | 0.13 | -0.12 | -0.11 | -0.11 | -0.30 | 0.23 | 0.11 |
| H4P_AUC | 0.16 | **.51**^**^ | -0.32 | 0.00 | -0.21 | 0.04 | -0.10 | -0.15 |
| H3P_AUC | -0.01 | -0.05 | 0.22 | 0.26 | 0.15 | 0.27 | **-.43**^*^ | -0.37 |
| H2P_AUC | -0.15 | -0.17 | 0.34 | 0.35 | 0.26 | 0.39 | -0.14 | -0.08 |
| H1P_AUC | -0.22 | -0.27 | 0.17 | 0.29 | 0.25 | 0.29 | 0.27 | 0.31 |
| TRLZ_AUC | -0.06 | -0.20 | **.61**^**^ | **.57**^**^ | 0.12 | 0.25 | -0.20 | -0.24 |
| LDLZ_AUC | -0.11 | -0.23 | -0.09 | 0.31 | **-.45**^*^ | 0.01 | -0.35 | -0.37 |
| HDLZ_AUC | -0.04 | -0.13 | 0.02 | 0.17 | **-.43**^*^ | -0.23 | **-.40**^*^ | -0.35 |
| NTG_AUC | -0.05 | -0.15 | 0.33 | 0.22 | 0.25 | 0.24 | -0.32 | -0.37 |
| NTC_AUC | -0.13 | -0.04 | -0.21 | -0.18 | -0.19 | -0.06 | **-.43**^*^ | **-.49**^*^ |
| NTRLTG_AUC | -0.05 | -0.16 | 0.36 | 0.24 | 0.27 | 0.26 | -0.29 | -0.35 |
| NTRLC_AUC | -0.07 | -0.16 | 0.21 | 0.13 | 0.17 | 0.14 | -0.36 | **-.41**^*^ |
| NLDLC_AUC | -0.14 | 0.05 | **-.42**^*^ | -0.39 | -0.27 | -0.16 | -0.26 | -0.32 |
| NHDLC_AUC | -0.09 | -0.05 | -0.08 | 0.25 | -0.08 | 0.05 | -0.34 | -0.28 |
| ApoB_AUC | -0.21 | -0.08 | -0.18 | -0.23 | -0.11 | -0.07 | -0.36 | -0.38 |
| ApoA1_AUC | -0.13 | -0.11 | 0.21 | 0.38 | 0.07 | 0.27 | -0.30 | -0.21 |
| BCAA_AUC | -0.03 | -0.25 | 0.33 | 0.14 | 0.08 | 0.03 | -0.22 | -0.19 |
| Val_AUC | 0.03 | -0.18 | 0.37 | 0.12 | -0.04 | -0.12 | -0.19 | -0.35 |
| Leu_AUC | -0.11 | -0.29 | 0.25 | 0.08 | 0.18 | 0.15 | -0.07 | 0.00 |
| Ileu_AUC | 0.08 | -0.16 | **.55**^**^ | 0.25 | 0.15 | 0.01 | -0.16 | -0.23 |
| Ala_AUC | **.46**^*^ | 0.32 | **.56**^**^ | 0.20 | 0.23 | 0.24 | -0.13 | -0.19 |
| Glu_AUC | 0.15 | -0.13 | **.46**^*^ | 0.13 | -0.06 | -0.26 | **-.45**^*^ | **-.46**^*^ |
| Ctr_AUC | -0.07 | -0.20 | 0.32 | 0.33 | -0.02 | 0.11 | -0.21 | -0.19 |
| KetBod_AUC | 0.01 | -0.10 | **.47**^*^ | **.43**^*^ | -0.13 | 0.06 | -0.19 | 0.26 |
| B-HB_AUC | -0.14 | -0.13 | 0.34 | 0.39 | -0.10 | 0.08 | -0.11 | 0.28 |
| AcAc_AUC | 0.27 | 0.23 | 0.25 | 0.21 | -0.05 | -0.01 | 0.12 | 0.08 |
| Acetone_AUC | 0.27 | 0.11 | **.51**^**^ | **.55**^**^ | -0.20 | -0.11 | **-.45**^*^ | -0.23 |
| LPIR_AUC | 0.08 | -0.15 | **.64**^**^ | **.45**^*^ | **.44**^*^ | **.40**^*^ | -0.08 | -0.12 |
| DRI_AUC | 0.03 | -0.26 | **.41**^*^ | 0.18 | 0.32 | 0.21 | -0.14 | -0.09 |
| GlycA_AUC | 0.03 | 0.02 | 0.05 | 0.11 | 0.02 | 0.24 | -0.08 | -0.03 |
| a-HB_AUC | 0.23 | 0.01 | 0.33 | 0.31 | 0.12 | 0.07 | -0.10 | -0.12 |
| Gly_AUC | 0.27 | 0.27 | -0.19 | -0.25 | 0.03 | 0.07 | -0.01 | 0.05 |
| Lactate_AUC | 0.33 | 0.15 | **.55**^**^ | **.45**^*^ | 0.33 | 0.31 | -0.19 | -0.20 |
| Pyruvate_AUC | **.51**^**^ | **.51**^**^ | 0.14 | 0.21 | -0.15 | -0.18 | -0.14 | -0.18 |
| IVX_AUC | -0.06 | 0.07 | -0.30 | -0.22 | -0.38 | -0.29 | -0.02 | -0.04 |
| IVF_AUC | -0.05 | 0.07 | -0.31 | -0.22 | **-.38**^*^ | -0.32 | -0.07 | -0.06 |
| IVM_AUC | -0.02 | 0.07 | -0.27 | -0.22 | -0.35 | -0.27 | -0.03 | -0.03 |
| MMX_AUC | 0.09 | -0.01 | 0.36 | **.48**^*^ | -0.25 | 0.01 | -0.29 | -0.25 |
| MMXF_AUC | -0.01 | -0.10 | 0.31 | **.52**^**^ | -0.19 | 0.11 | -0.19 | -0.18 |
| MMXM_AUC | 0.12 | 0.05 | 0.31 | **.42**^*^ | -0.27 | -0.06 | -0.30 | -0.28 |
| MVX_AUC | 0.01 | 0.07 | -0.18 | -0.04 | **-.43**^*^ | -0.25 | -0.05 | -0.09 |
| MVXF_AUC | 0.03 | 0.04 | -0.16 | 0.00 | **-.44**^*^ | -0.24 | -0.10 | -0.08 |
| MVXM_AUC | 0.04 | 0.09 | -0.18 | -0.06 | **-.39**^*^ | -0.24 | -0.05 | -0.08 |

**. Correlation is significant at the 0.01 level (2-tailed)

*. Correlation is significant at the 0.05 level (2-tailed)

**Supplementary Table 4:** Spearman Correlation matrix of C-peptide, GDF15 (total and h-specific) and GIP postprandial iAUCs with the postprandial iAUCs of metabolipidomic variables; unadjusted and adjusted for age and BMI.

|  | C-peptide iAUC | C-peptide iAUC adjusted | Total GDF15 iAUC | Total GDF15 iAUC adjusted | H-specific GDF15 iAUC | H-specific GDF15 iAUC adjusted | GIP iAUC | GIP iAUC adjusted |
| --- | --- | --- | --- | --- | --- | --- | --- | --- |
| TRLP_iAUC | 0.09 | 0.28 | 0.22 | **.40**^*^ | 0.05 | 0.28 | -0.07 | -0.03 |
| VL-TRLP_iAUC | -0.22 | -0.19 | 0.03 | 0.12 | -0.13 | 0.14 | 0.05 | 0.00 |
| L-TRLP_iAUC | 0.33 | **.46**^*^ | 0.18 | 0.31 | 0.33 | **.58**^**^ | 0.09 | 0.15 |
| M-TRLP_iAUC | -0.03 | 0.03 | 0.29 | 0.22 | **.38**^*^ | **.69**^**^ | 0.13 | 0.00 |
| S-TRLP_iAUC | 0.11 | 0.33 | 0.16 | 0.30 | 0.04 | 0.15 | 0.12 | 0.15 |
| VS-TRLP_iAUC | 0.11 | 0.13 | 0.15 | 0.27 | 0.10 | 0.23 | -0.11 | -0.19 |
| cLDLP_iAUC | 0.18 | 0.27 | 0.34 | **.51**^**^ | **.42**^*^ | **.54**^**^ | 0.07 | 0.21 |
| L-cLDLP_iAUC | -0.04 | 0.22 | **.58**^**^ | **.63**^**^ | 0.29 | **.50**^*^ | 0.18 | 0.17 |
| M-cLDLP_iAUC | 0.13 | -0.11 | 0.19 | 0.21 | 0.19 | 0.02 | **.40**^*^ | **.44**^*^ |
| S-cLDLP_iAUC | 0.16 | 0.32 | 0.07 | 0.15 | 0.17 | **.42**^*^ | -0.03 | -0.08 |
| cHDLP_iAUC | 0.25 | 0.23 | **.58**^**^ | **.61**^**^ | 0.35 | **.40**^*^ | 0.13 | 0.27 |
| L-cHDLP_iAUC | 0.24 | 0.30 | **.41**^*^ | **.49**^*^ | 0.24 | **.40**^*^ | 0.04 | 0.22 |
| M-cHDLP_iAUC | 0.18 | 0.38 | 0.37 | **.56**^**^ | 0.09 | **.48**^*^ | 0.09 | 0.11 |
| S-cHDLP_iAUC | 0.03 | 0.07 | **.38**^*^ | **.55**^**^ | 0.34 | 0.27 | 0.30 | 0.33 |
| H7P_iAUC | -0.06 | 0.15 | **.44**^*^ | **.47**^*^ | 0.32 | **.42**^*^ | -0.06 | 0.09 |
| H6P_iAUC | 0.25 | 0.27 | 0.27 | 0.22 | 0.17 | 0.10 | 0.25 | 0.36 |
| H5P_iAUC | 0.11 | 0.09 | 0.08 | 0.20 | 0.04 | 0.22 | 0.05 | -0.11 |
| H4P_iAUC | 0.14 | 0.21 | **.54**^**^ | **.43**^*^ | 0.30 | 0.26 | 0.11 | 0.14 |
| H3P_iAUC | 0.11 | 0.28 | 0.04 | 0.31 | -0.08 | 0.35 | 0.02 | 0.01 |
| H2P_iAUC | 0.11 | 0.11 | **.47**^*^ | **.65**^**^ | 0.29 | 0.30 | 0.18 | 0.22 |
| H1P_iAUC | -0.09 | -0.13 | -0.15 | -0.12 | 0.10 | -0.01 | 0.35 | **.48**^*^ |
| TRLZ_iAUC | 0.05 | 0.20 | **.56**^**^ | **.58**^**^ | **.51**^**^ | **.62**^**^ | 0.17 | 0.22 |
| LDLZ_iAUC | -0.09 | 0.26 | **.59**^**^ | **.56**^**^ | 0.36 | **.52**^**^ | 0.36 | 0.25 |
| HDLZ_iAUC | 0.01 | 0.25 | 0.33 | **.53**^**^ | 0.19 | **.51**^**^ | 0.13 | 0.23 |
| NTG_iAUC | 0.27 | 0.32 | **.51**^**^ | **.58**^**^ | **.42**^*^ | **.74**^**^ | 0.03 | 0.11 |
| NTC_iAUC | 0.28 | 0.28 | **.59**^**^ | **.58**^**^ | 0.28 | **.52**^**^ | -0.01 | 0.20 |
| NTRLTG_iAUC | 0.23 | 0.30 | **.49**^**^ | **.57**^**^ | **.42**^*^ | **.82**^**^ | -0.02 | 0.05 |
| NTRLC_iAUC | 0.11 | 0.35 | 0.22 | **.50**^*^ | 0.28 | **.56**^**^ | -0.08 | -0.02 |
| NLDLC_iAUC | 0.03 | 0.25 | **.45**^*^ | **.55**^**^ | **.42**^*^ | **.52**^**^ | 0.21 | 0.23 |
| NHDLC_iAUC | 0.25 | 0.28 | **.64**^**^ | **.59**^**^ | 0.31 | **.43**^*^ | 0.04 | 0.23 |
| ApoB_iAUC | 0.20 | 0.27 | 0.28 | **.52**^**^ | 0.31 | **.51**^**^ | -0.03 | 0.20 |
| ApoA1_iAUC | 0.27 | 0.27 | **.61**^**^ | **.61**^**^ | 0.33 | **.41**^*^ | 0.09 | 0.25 |
| BCAA_iAUC | 0.06 | 0.24 | 0.34 | **.54**^**^ | 0.12 | 0.34 | 0.13 | 0.21 |
| Val_iAUC | 0.19 | 0.28 | **.44**^*^ | **.53**^**^ | 0.27 | 0.37 | 0.22 | 0.24 |
| Leu_iAUC | 0.11 | 0.22 | 0.24 | **.52**^**^ | -0.04 | 0.28 | 0.07 | 0.17 |
| Ileu_iAUC | 0.00 | 0.04 | 0.28 | **.42**^*^ | 0.08 | 0.25 | 0.18 | 0.11 |
| Ala_iAUC | 0.14 | 0.19 | **.41**^*^ | **.49**^*^ | **.57**^**^ | **.75**^**^ | 0.28 | 0.17 |
| Glu_iAUC | 0.03 | -0.09 | 0.13 | 0.13 | 0.28 | 0.39 | -0.11 | -0.20 |
| Ctr_iAUC | 0.07 | 0.23 | 0.34 | **.55**^**^ | 0.31 | **.62**^**^ | 0.05 | 0.12 |
| KetBod_iAUC | 0.29 | 0.20 | 0.20 | 0.13 | 0.28 | 0.08 | 0.18 | -0.09 |
| B-HB_iAUC | 0.28 | 0.18 | 0.08 | 0.05 | 0.15 | 0.03 | 0.04 | -0.18 |
| AcAc_iAUC | **.41**^*^ | 0.28 | **.45**^*^ | 0.26 | 0.32 | 0.11 | 0.33 | 0.35 |
| Acetone_iAUC | 0.02 | -0.21 | 0.29 | 0.34 | **.47**^*^ | 0.32 | 0.14 | 0.12 |
| LPIR_iAUC | 0.14 | 0.17 | 0.27 | **.41**^*^ | 0.38 | **.76**^**^ | 0.14 | 0.07 |
| DRI_iAUC | 0.21 | 0.17 | 0.29 | **.51**^**^ | 0.08 | 0.39 | 0.14 | 0.11 |
| GlycA_iAUC | 0.24 | 0.33 | **.68**^**^ | **.66**^**^ | 0.36 | **.46**^*^ | 0.17 | 0.27 |
| a-HB_iAUC | 0.20 | 0.20 | 0.16 | 0.15 | 0.10 | 0.22 | 0.32 | 0.32 |
| Gly_iAUC | 0.18 | 0.29 | **.48**^*^ | **.56**^**^ | 0.29 | 0.37 | 0.02 | 0.18 |
| Lactate_iAUC | 0.19 | 0.31 | **.43**^*^ | **.55**^**^ | **.55**^**^ | **.61**^**^ | -0.04 | 0.00 |
| Pyruvate_iAUC | 0.27 | 0.21 | 0.17 | 0.29 | 0.33 | 0.37 | -0.15 | -0.20 |
| IVX_iAUC | 0.00 | 0.26 | **.43**^*^ | **.46**^*^ | 0.22 | **.46**^*^ | 0.16 | 0.19 |
| IVF_iAUC | -0.04 | 0.25 | **.39**^*^ | **.45**^*^ | 0.23 | **.47**^*^ | 0.13 | 0.17 |
| IVM_iAUC | 0.00 | 0.27 | **.44**^*^ | **.47**^*^ | 0.23 | **.45**^*^ | 0.19 | 0.20 |
| MMX_iAUC | 0.03 | 0.27 | 0.30 | **.56**^**^ | **.52**^**^ | **.60**^**^ | 0.17 | 0.19 |
| MMXF_iAUC | 0.00 | 0.27 | 0.20 | **.57**^**^ | **.46**^*^ | **.59**^**^ | 0.10 | 0.17 |
| MMXM_iAUC | 0.01 | 0.24 | 0.33 | **.53**^**^ | **.56**^**^ | **.61**^**^ | 0.17 | 0.20 |
| MVX_iAUC | 0.08 | 0.27 | 0.36 | **.49**^*^ | **.39**^*^ | **.51**^**^ | 0.15 | 0.19 |
| MVXF_iAUC | 0.02 | 0.26 | 0.26 | **.49**^*^ | 0.36 | **.53**^**^ | 0.08 | 0.16 |
| MVXM_iAUC | 0.07 | 0.26 | **.42**^*^ | **.49**^*^ | **.40**^*^ | **.50**^*^ | 0.20 | 0.20 |

**. Correlation is significant at the 0.01 level (2-tailed)

*. Correlation is significant at the 0.05 level (2-tailed)

**Supplementary Table 5:** Exploratory Spearman correlation matrix of C-peptide, GDF15 (total and h-specific) and GIP with basic anthropometric and biochemical variables

|  | Fasting GIP | Fasting total GDF15 | Fasting H-Specific GDF15 | Fasting C-peptide |
| --- | --- | --- | --- | --- |
| Weight | -0.02 | 0.13 | -0.03 | **.64^**^** |
| BMI | -0.03 | 0.10 | 0.00 | **.67^**^** |
| Waist | 0.00 | -0.05 | -0.15 | **.33^*^** |
| Hip | 0.00 | -0.09 | -0.10 | **.59^**^** |
| Total cholesterol | 0.04 | -0.01 | 0.02 | -0.24 |
| LDL-c | -0.01 | 0.23 | 0.15 | -0.01 |
| HDL-c | -0.05 | -0.27 | -0.09 | -0.25 |
| Triglycerides | -0.02 | **.30^*^** | **.31^*^** | -0.14 |
| AST | -0.08 | **.57^**^** | 0.10 | 0.08 |
| ALT | -0.18 | -0.06 | -0.16 | **.31^*^** |
| GGT | 0.04 | 0.10 | -0.09 | 0.27 |
| HbA1C | -0.05 | -0.08 | 0.01 | -0.04 |
| Fasting glucose | -0.17 | **.36^*^** | 0.11 | 0.15 |

**. Correlation is significant at the 0.01 level (2-tailed)

*. Correlation is significant at the 0.05 level (2-tailed)
